# SIRT3 deficiency delays diabetic skin wound healing via oxidative stress and necroptosis enhancement

**DOI:** 10.1111/jcmm.15100

**Published:** 2020-03-02

**Authors:** Shengju Yang, Mengting Xu, Guoliang Meng, Yan Lu

**Affiliations:** ^1^ Department of Dermatology The First Affiliated Hospital of Nanjing Medical University Nanjing China; ^2^ Department of Dermatology Affiliated Hospital of Nantong University Nantong China; ^3^ Department of Pharmacology School of Pharmacy Nantong University Nantong China

**Keywords:** diabetes mellitus, necroptosis, oxidative stress, Sirtuin 3, skin fibroblasts, skin wound healing

## Abstract

Sirtuin 3 (SIRT3) plays a vital role in several dermatological diseases. However, the role and detailed mechanism of SIRT3 in diabetic wound healing are unknown well yet. To explore possible involvement of SIRT3 and necroptosis in diabetic skin wound healing, SIRT3 knockout (KO) mice and 129S1/SvImJ wild‐type (WT) mice were injected with streptozotocin (STZ), and mice skin fibroblasts were exposed to high glucose (HG). It was found that SIRT3 expression decreased in the skin of diabetic patients. SIRT3 deficiency delayed healing rate, reduced blood supply and vascular endothelial growth factor expression, promoted superoxide production, increased malondialdehyde (MDA) levels, decreased total antioxidant capacity (T‐AOC), reduced superoxide dismutase (SOD) activity and aggravated ultrastructure disorder in skin wound of diabetic mice. SIRT3 deficiency inhibited mice skin fibroblasts migration with HG stimulation, which was restored by SIRT3 overexpression. SIRT3 deficiency also suppressed α‐smooth muscle actin (α‐SMA) expression, enhanced superoxide production but decreased mitochondrial membrane potential with HG stimulation after scratch. SIRT3 deficiency further elevated receptor‐interacting protein kinase 3 (RIPK3), RIPK1 and caspase 3 expression both in vitro and in vivo. Collectively, SIRT3 deficiency delayed skin wound healing in diabetes, the mechanism might be related to impaired mitochondria function, enhanced oxidative stress and increased necroptosis. This may provide a novel therapeutic target to accelerate diabetic skin wound healing.

## 1 INTRODUCTION

Diabetes mellitus (DM) is a common metabolic disease characterized by hyperglycaemia. DM suffering for a long‐term usually leads to chronic damage on eyes, kidneys, heart, blood vessels and nerves, resulting in various complications and even death.^1^ It is also worth noting that patients with DM may have decreased immune function, uncontrollable infection and delayed skin wound healing to cause or aggravate the occurrence and progress of diabetic foot or gangrene.^2^, ^3^, ^4^ Therefore, to clarify the pathogenesis of delayed wound healing in diabetic skin will be conducive to find out novel strategies to accelerate wound healing process.

Previous studies have verified that moderate oxidative stress is essential for skin wound healing.^5^ On the contrary, excessive oxidative stress delays skin wound healing especially in DM by inhibiting angiogenesis, regulating extracellular matrix deposition and disordering cytokine activity.^6^, ^7^ However, the role and the exact mechanism of oxidative stress in delayed diabetic skin wound healing remain unclear.

Sirtuin 3 (SIRT3), as one type of histone deacetylases, offers great influence on oxidative stress to regulate inflammation, hypertension, hyperglycaemia and so on.^8^, ^9^, ^10^ SIRT3 overexpression alleviated oxidative stress and inhibited keratinocytes differentiation.^11^ One group showed that there was less expression of SIRT3 in skin tissue samples of systemic scleroderma patients than that of healthy controls.^12^ Some studies demonstrated that SIRT3 was closely related to the occurrence and development of cutaneous melanoma.^13^ These studies suggested that SIRT3 might play a vital role in the skin system, but whether SIRT3 is crucial in skin wound healing of DM is unknown well yet.

SIRT3 played an important role in energy metabolism and oxidative stress. Necroptosis, a new type of cell death, was able to be triggered by reactive oxygen species (ROS).^14^ At present, it is believed that receptor‐interacting protein kinase 3 (RIPK3) is one critical molecule to mediate necroptosis. RIPK3 binds to receptor‐interacting protein kinase 1 (RIPK1) and then activate each other to form a complex called necrosome, which combines with mixed lineage kinase domain‐like protein (MLKL) to accelerate cell necrosis and promote cell damage.^15^, ^16^, ^17^ RIPK3 and RIPK1 are regarded as the sensitive markers of necroptosis.^18^ There is evidence that necroptosis has been participated in the pathogenesis of several diseases, including cardiovascular diseases, neurological disorders, digestive system diseases and so on.^16^, ^19^, ^20^ Therefore, the possible involvement of necroptosis in diabetic skin wound was also investigated in our present study.

To explore the role and the mechanism of SIRT3 in skin wound healing, SIRT3 knockout (KO) mice and 129S1/SvImJ wild‐type (WT) mice were injected with streptozotocin (STZ), and mice skin fibroblasts were exposed to high glucose in our present study. The wound healing process and cell migration rate were assessed, and the possible mechanism was investigated. It is beneficial to provide novel ideas for clinical prevention and treatment of diabetic skin wound delay.

## 2 MATERIALS AND METHODS

### 2.1 Collection of human skin samples

Discarded skin samples, from 6 diabetic patients and 6 normoglycemic patients who needed surgery, were collected for further histological examination, Western blot and PCR analysis.^21^ All normoglycemic patients, without general infection or cardiovascular or renal diseases, had normal fasting blood glucose and glycosylated haemoglobin.^21^ The study protocol was approved by the ethics committee of Affiliated Hospital of Nantong University (2017‐L050). Written informed consent was obtained from all patients. The research was carried out according to the World Medical Association Declaration of Helsinki.

### 2.2 Animal treatment

129S1/SvImJ (WT) and SIRT3 KO mice at 8 week old were randomly and intraperitoneally injected with STZ (60 mg/kg) in freshly prepared citrate buffer solution (0.1 mol/L, pH 4.5) or citrate buffer solution (0.1 mol/L, pH 4.5) once daily. After five consecutive days of administration, fasting blood glucose (FBG) was measured from tail vein with a One‐Touch blood glucosemeter (Johnson, USA). Mice with FBG more than 16.7 mmol/L were confirmed as diabetic mice and used for further experiments.^22^

Twelve weeks later, both diabetic mice (DM group) and non‐diabetic mice (control group) were intraperitoneally injected with ketamine (100 mg/kg) and xylazine (5 mg/kg) to induce anaesthesia. After reflex to toe‐pinching disappeared, a round skin wound about 6 mm diameter with depth into the dermis was conducted by a biopsy perforator at the back spine.

All experimental procedures were approved by Ethics Committee of Nantong University, and all animals received human care in strict accordance with the National Institutes of Health guidelines.

### 2.3 Wound observation and blood perfusion examination

The photographs of the mice skin wound were recorded to assess the healing degree, which were represented as the percentage of wound area to original size at different time after surgery.

Then, after anaesthesia with 1.5% isoflurane, blood perfusion graph around the skin trauma of mice was recorded with laser Doppler (Perimed, PSI, Perimed) immediately after trauma surgery (0 d), and 1st day (1 d), 3rd day (3 d), 5th day (5 d) and 7th day (7 d) after surgery. The average blood intensity was automatically analysed by the software.^23^

### 2.4 Haematoxylin and eosin (HE) staining

On the 7th day after surgery, the non‐healing mice wound tissue and the mice skin tissue within 2 mm from the edge of wound were collected. Tissues were fixed with 4% paraformaldehyde for 24 hours followed by HE staining.

### 2.5 ROS measurement

After incubation with dihydroethidium (DHE, 2 μmol/L, Beyotime, Shanghai, China) at 37°C for 30 min, the global superoxide production of mice skin samples or mice skin fibroblasts were measured as DHE fluorescence intensity. Mitochondrial ROS of the skin fibroblasts was detected with Mito‐tracker Green (100 nmol/L, Beyotime) and MitoSOX (5 μmol/L, YEASEN) incubation at 37°C for 20 minutes. The fluorescence intensity was assessed with a laser confocal microscope (Leica).

According to the instructions of the commercial kits (Beyotime), malondialdehyde (MDA) level of the mice skin samples was detected with thiobarbituric acid method. Total antioxidant capacity (T‐AOC) was assessed with 2,2'‐azino‐bis (3‐ethylbenzthi‐azoline‐6‐sulphonic acid, ABTS) method. Superoxide dismutase (SOD) activity was measured with the WST‐8 [5‐(2,4‐Disulfophenyl)‐3‐(2‐methoxy‐4‐nitrophenyl)‐2‐(4‐nitrophenyl)‐2H‐ tetrazolium] method.

### 2.6 Ultrastructure measurement

Mice skin wound samples were cut into pieces of 1 mm^3^ and fixed with 4% glutaraldehyde at 4°C and 1% osmium tetroxide at room temperature successively. The samples were dehydrated, infiltrated, embedded with Epon812, and cut into ultrathin sections of 60‐80 nm followed by staining with uranyl acetate and lead citrate. The ultrastructure was examined with transmission electron microscope (HT7700, HITACH, Japan).

### 2.7 Primary skin fibroblast cell culture

The skin from 3‐day‐old WT mice or SIRT3 KO mice was cut into small pieces (1 mm^3^) after removing epidermis and surrounding fat. The primary mice skin fibroblasts were cultured in Dulbecco's modified Eagle's medium (DMEM) with 10% foetal bovine serum (FBS, Gibco) containing normal glucose (5.5 mmol/L, NG) or high glucose (30 mmol/L, HG). The mice skin fibroblasts of 3rd to 6th passage were subjected to further experiments.

### 2.8 SIRT3 adenovirus infection

Recombinant adenovirus carrying mice SIRT3 gene (1 × 10^11^ PFU/mL) or Vector (1 × 10^11^ PFU/mL, Hanbio Biotechnology Co, Ltd) with MOI value of 100 were infected into the mice skin fibroblasts. After 4 hours, the adenovirus solution was washed out. Then, the cells were subjected to scratch migration assay.

### 2.9 Scratch migration assay

Pipette tips of 10 µL were applied to induce a scratch in mice skin fibroblasts. The shedding cells were washed with PBS. After scratch for 0, 4, 8 and 12 hours, cell closures were recorded by photograph. The migration rate of the scratched cells was statistically analysed as ratio of migrated area to original area.

### 2.10 Mitochondrial membrane potential (Δψm) detection

After incubation with JC‐1 staining solution (Beyotime, Shanghai, China) for 20 minutes, the fluorescence intensity, respecting Δψm, in the mice skin fibroblasts was assessed with a laser confocal microscope at 495/519 nm wavelengths for monomer and 550/570 nm wavelengths for J‐aggregates, respectively.

### 2.11 Immunohistochemistry or immunofluorescent staining

After incubation with SIRT3, α‐smooth muscle actin (α‐SMA), PIPK1 or PIPK3 (1:50) antibodies overnight at 4°C, the section of human skin sample or mice skin fibroblasts was incubated with affinity‐purified biotinylated IgG, or Alexa Fluor 488 conjugated IgG (1:500; Beyotime) for 2 hours.

### 2.12 Quantitative real‐time PCR

Total RNA was extracted with Trizol from human or mice skin samples as well as mice skin fibroblasts. Then, RNA sample and reaction buffer were mixed for reverse transcription with the procedure: incubation at 37°C for 15 min, 85°C for 5 seconds and preservation at 4°C. Reversed cDNA was mixed with the SYBR Green qPCR mixture (Takara, Otsu, Shiga, Japan) for further amplification (ABI). The primer sequences of human‐SIRT3 mRNA (F, 5'‐TGCAGAAGTAGCAGTTCAGTG‐3' and R, 5'‐GCTTCCTCTAGTGACACTGTTAG‐3'), mice‐vascular endothelial growth factor (VEGF) mRNA (F, 5'‐ F 5'‐CGCCGCAGGAGACAAACCGAT‐3' and R, 5'‐ACCCGTCCATGAGCTCGGCT‐3'), mice‐α‐SMA mRNA (F, 5'‐TCCCTGGAGAAGAGCTACGAACT‐3' and R, 5'‐AAGCGTTCGTTTCCAATGGT‐3') as well as housekeeping 18S mRNA (F, 5'‐AGTCCCTGCCCTTTGTACACA‐3' and R, 5'‐CGATCCGAGGGCCTCACTA‐3') were synthesized by Sangon Biotech Co., Ltd (Shanghai, China). PCR was performed three times, and the relative mRNA level was calculated by Comparative Delta‐delta Cycle threshold Method.

### 2.13 Western blot

The proteins, extracted from human or mice skin samples as well as mice skin fibroblasts, were separated by sodium dodecyl sulphate‐polyacrylamide gel electrophoresis (SDS‐PAGE), and transferred to a polyvinylidene fluoride (PVDF) membrane. Then, the membranes were immerged into 5% milk without fat to block for 2 hours. Next, they were incubated with primary antibodies including anti‐SIRT3, caspase 3, MLKL, p‐MLKL, RIPK1 (1:1000, Cell Signaling Technology), anti‐RIPK3 (1:1000, Novusbio), anti‐α‐SMA (1:1000, Bosterbio), anti‐GAPDH (1:5000, Sigma‐Aldrich) and anti‐β‐tubulin (1:3000, CMCTAG) at 4°C for more than 12 hours. Horseradish peroxidase (HRP)‐conjugated IgG (Beyotime) or enhanced chemiluminescence (ECL, Thermo Fisher Scientific Inc) was applied to visualize the protein bands.

### 2.14 Statistical analysis

The data were expressed as mean ± Standard Error of the Mean (SEM), which were analysed by one‐way ANOVA followed by Bonferroni *post hoc* test. *P*‐values lower than 0.05 was regarded as significant difference.

## 3 RESULTS

### 3.1 SIRT3 expression decreased in skin of diabetic patients

**FIGURE 1:**
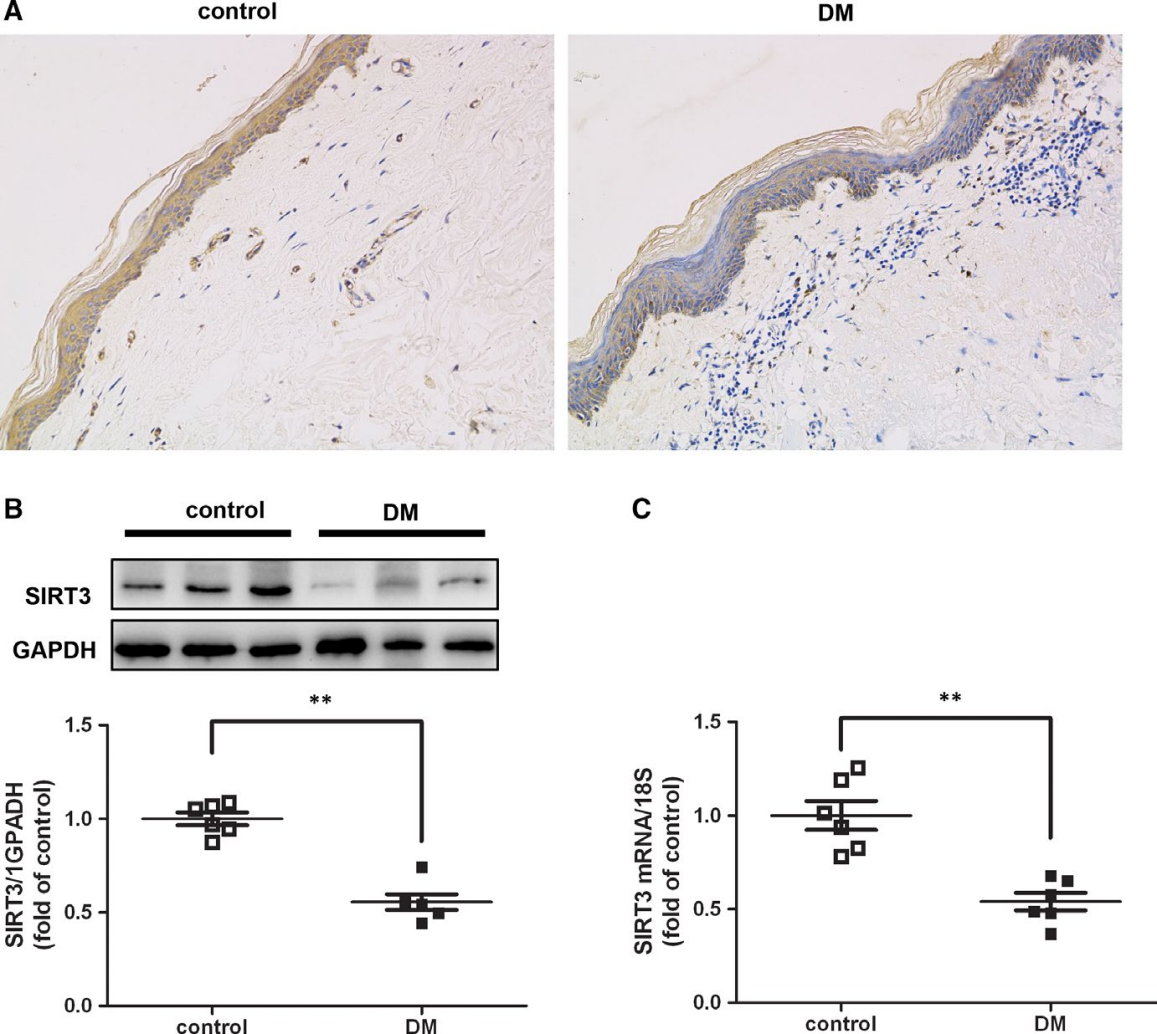
SIRT3 expression decreased in skin of diabetic patients. Skin samples were collected from diabetic or normoglycemic patients. A, SIRT3 expression was measured by immunohistochemistry staining (40×). B and C, SIRT3 expression was measured by Western blot and real‐time PCR. ^**^ *P* < .01 *vs* control, n = 6

Compared to control normoglycemic patients, all skin from diabetic patients exhibited lower expression of SIRT3 than normoglycemic skin by immunohistochemistry (Figure 1A), Western blot (Figure 1B) and real‐time PCR (Figure 1C). It was suggested that SIRT3 pathway was impaired in the skin tissues of patients with diabetes. Consequently, SIRT3 KO mice were used to investigate the role of SIRT3 in wound healing with DM in vitro and in vivo.

### 3.2 SIRT3 deficiency delayed the healing rate of skin wound in diabetic mice

**FIGURE 2:**
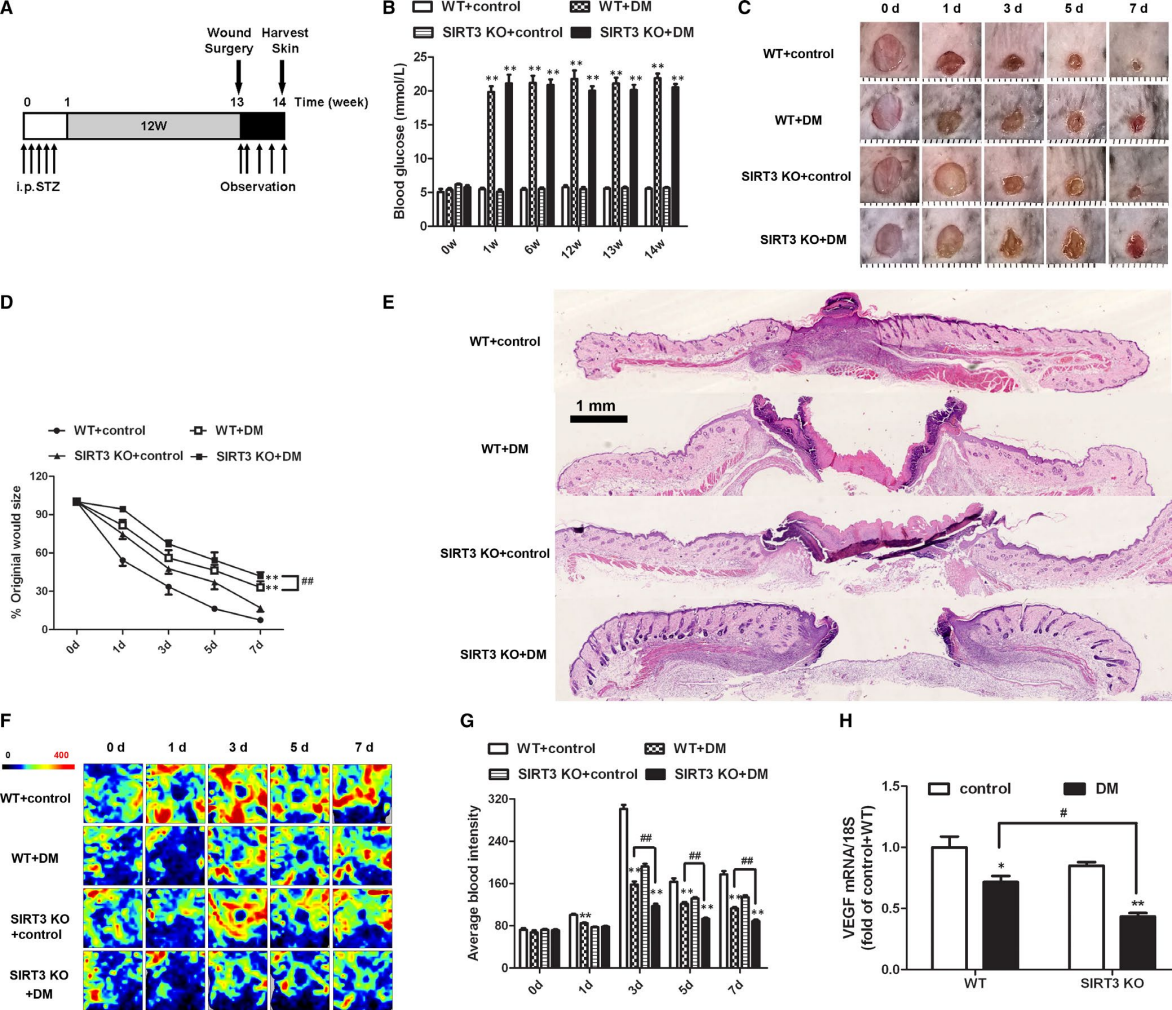
SIRT3 deficiency delayed the healing rate and reduced blood supply of skin wound in diabetic mice. A, Timeline for treatment, surgery, observation and wound healing assessment in 129S1/SvImJ (WT) and SIRT3 KO mice. B, Fasting blood glucose concentration was measured. C, The photographs of the skin wound were recorded at different time after surgery. D, The percentage of wound area to original size was calculated. E, Tissues from the skin wound on 7th day were stained with HE and photographed. F, Blood perfusion graph around the skin wound of mice was recorded according to the colour of perfusion graph at different time after surgery, which displayed redder if there was richer blood perfusion. G, The average blood intensity was quantitatively analysed during the recovery process. H, Vascular endothelial growth factor (VEGF) mRNA expression of the skin wound on 7th day was measured by real‐time PCR. *P < .05, **P < .01 *vs* non‐diabetic control group with the same genotype, ^#^ *P* < .05, ^##^ *P* < .01 vs DM of WT mice, n = 6

At the beginning of experiment, there was no significant difference on the level of FBG in all four groups. All the FBG values were more than 16.7 mmol/L in both WT mice and SIRT3 KO mice after STZ injection for 5 days, suggesting successful induction of DM in our study. The FBG kept above 16.7 mmol/L at the 6th, 12th, 13th and 14th week in STZ‐injected mice, suggesting high glucose was sustained during the whole experiments in diabetic mice (Figure 2A‐B).

Macroscopically, there were larger areas of skin wound in DM group than that in control mice with the same gene type. What is more, the area of the skin wound in DM of SIRT3 deficient mice was bigger than that in WT mice (Figure 2C‐D). HE staining also confirmed that there were greater areas of skin wound in DM especially with SIRT3 deficiency (Figure 2E). There data suggested that SIRT3 deficiency delayed the healing rate of skin wound in DM.

### 3.3 SIRT3 deficiency reduced blood supply around the skin wound in diabetic mice

To determine whether the difference in the speed of wound healing in respective groups was related to blood supply, blood perfusion was recorded in the process of skin wound healing. Blood flow during the recovery process was assessed according to the colour of perfusion graph, which displayed redder if there was richer blood perfusion. Our present study showed no significant difference in average blood intensity of skin wound among 4 groups immediately after the surgery. Compared to the control group, average blood intensity around the wound of diabetic mice was weaker than that of non‐diabetic mice with the same gene type on the 3rd, 5th and 7th day. What's more, blood flow around the skin trauma significantly decreased in DM of SIRT3 deficient mice compared with WT mice (Figure 2F‐G). Our studies also revealed that VEGF mRNA in the skin wound of diabetic mice was less than that of non‐diabetic mice on 7th day after surgery. Compared to WT mice with DM, VEGF mRNA expression in DM of SIRT3 KO mice remarkably decreased (Figure 2H).

### 3.4 SIRT3 deficiency heightened oxidative stress and aggravated ultrastructure disorder of skin wound in diabetic mice

**FIGURE 3:**
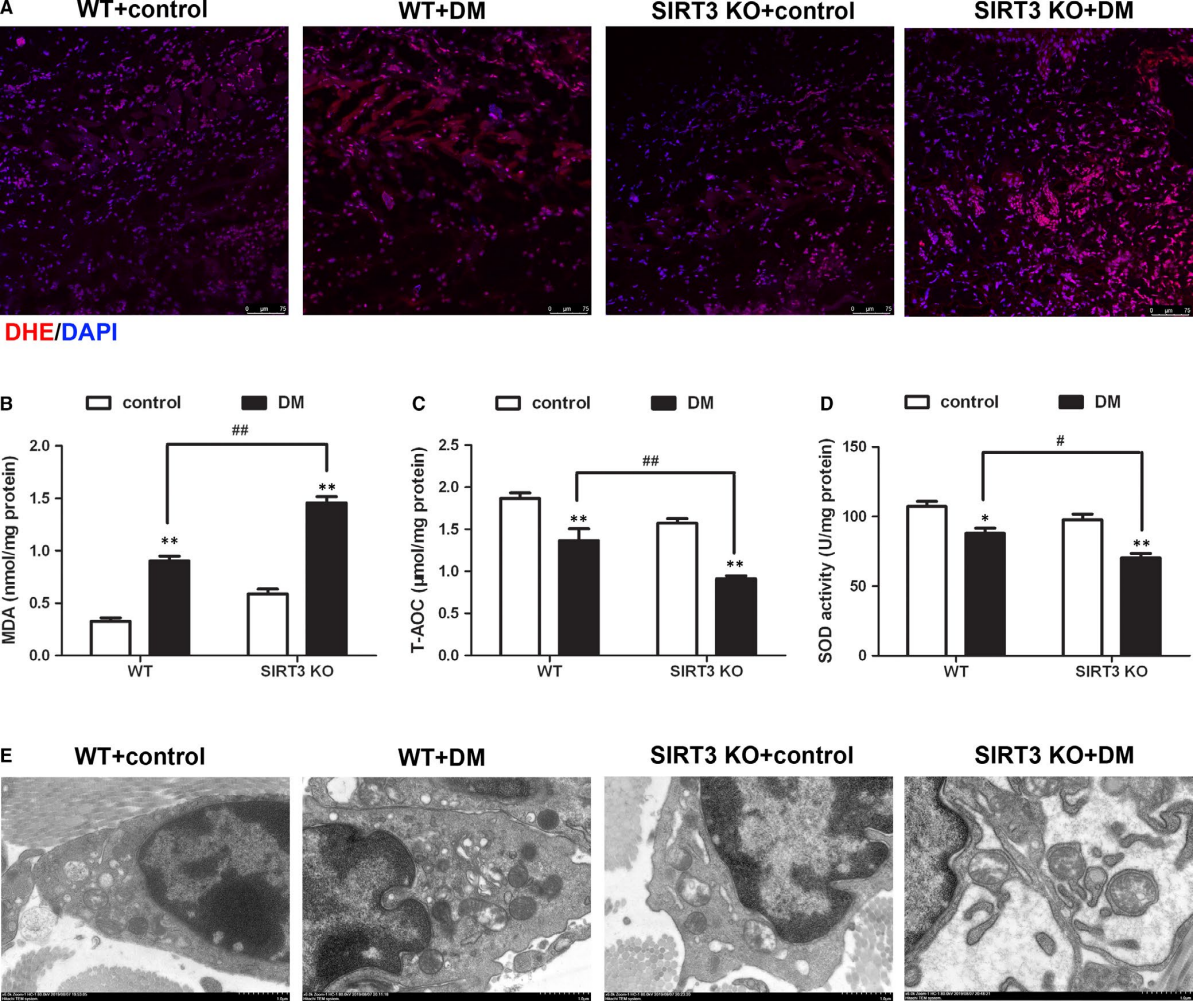
SIRT3 deficiency heightened oxidative stress and aggravated ultrastructure disorder of skin wound in diabetic mice. Skin samples around the wound were collected on 7th day after surgery. A, ROS production was measured with DHE fluorescent probe. Bar = 75 µm. B and D, Level of MDA, T‐AOC and SOD activity of skin wound was detected. E, Ultrastructure of the skin wound was examined with transmission electron microscope. Bar = 1 µm. ^*^ *P*< .05, ^**^ *P*< .01 *vs* non‐diabetic control group with the same genotype,^#^ *P*< .05, ^##^ *P* < .01 *vs* DM of WT mice, n = 6

Intensity of DHE staining of skin wound was enhanced in DM, which was further heightened in SIRT3 KO mice (Figure 3A). We also found that MDA level increased, while T‐AOC and SOD activity decreased in the skin wound of diabetic mice on 7th day after surgery. Compared to WT mice with DM, MDA level in DM of SIRT3 KO mice was remarkably enhanced but T‐AOC and SOD activity was further suppressed (Figure 3B‐D). These results suggested that SIRT3 deficiency promoted ROS production and oxidative damage in skin wound of mice with DM.

Transmission electron microscopy showed that the mitochondrial cristae in the skin of SIRT3 KO mice with diabetes became fewer, but fragmented and disordered. SIRT3 KO mice with diabetes exhibited mitochondria swelling with greater volume (Figure 3E).

### 3.5 SIRT3 deficiency promoted necroptosis of skin wound in diabetic mice

**FIGURE 4:**
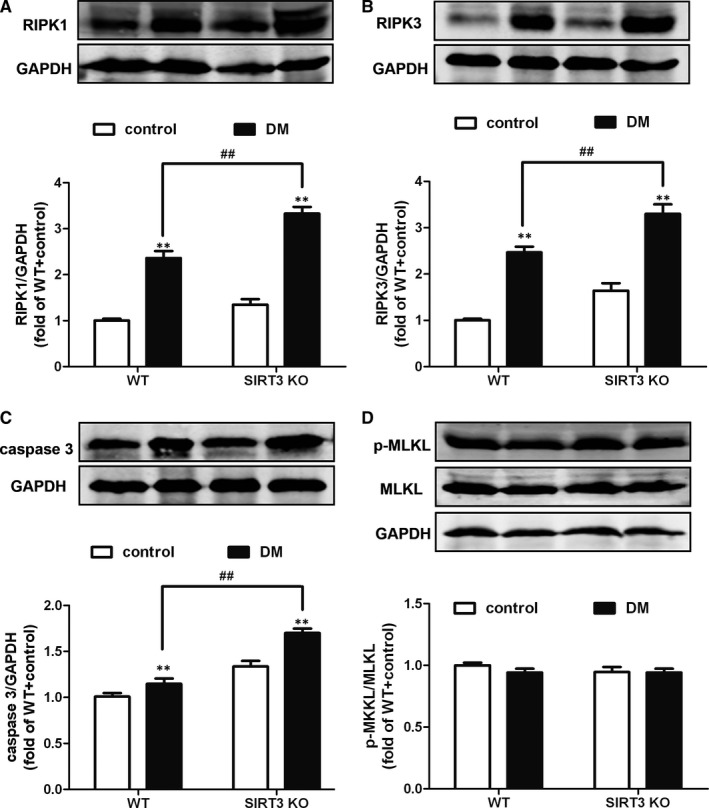
SIRT3 deficiency promoted necroptosis of skin wound in diabetic mice. The expression of RIPK1 (A), RIPK3 (B), caspase 3 (C), MLKL expression and phosphorylation (D) in the skin around the wound on 7th day after surgery was measured with Western blot. GAPDH was serviced as a housekeeping control. ^**^ *P* < .01 *vs* non‐diabetic control group with the same genotype, ^##^ *P* < .01 *vs* DM of WT mice, n = 6

Accumulation of ROS was a dominant factor to induce necroptosis,^24^, ^25^ which might impair the survival of cells and tissues around the wound area to delay healing. Expressions of RIPK1, RIPK3 and caspase 3 were regarded as sensitive and robust markers of necroptosis. Compared to the control group, the expression of RIPK1, RIPK3 and caspase 3 was notably increased in the skin wound of DM both in WT and SIRT3 KO mice (Figure 4A‐C). Moreover, compared to the DM group in WT mice, RIPK1, RIPK3 and caspase 3 expression in DM of SIRT3 KO mice remarkably increased (Figure 4A‐C), suggesting that SIRT3 played a protective role against necroptosis in skin wound of DM. Interestingly, the p‐MLKL expression showed no statistical significance between control and DM groups both in WT and SIRT3 KO mice (Figure 4D).

### 3.6 SIRT3 deficiency inhibited mice skin fibroblasts migration and proliferation with high‐glucose stimulation after scratch

**FIGURE 5:**
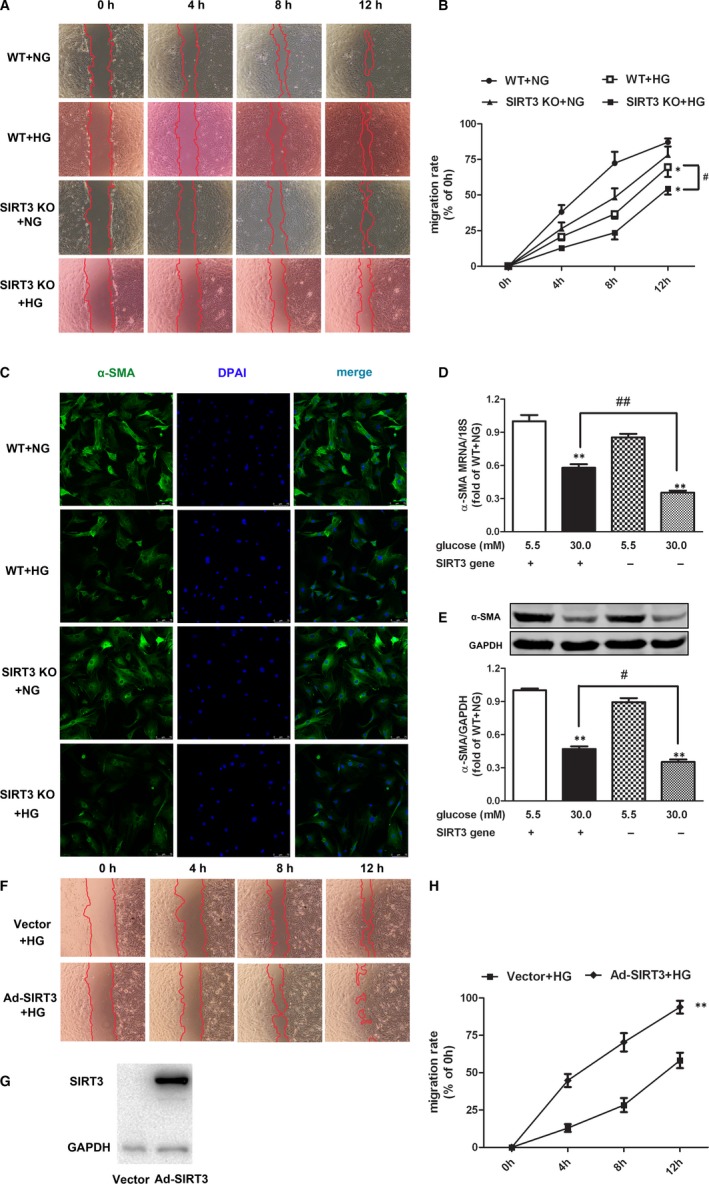
SIRT3 deficiency inhibited mice skin fibroblast migration and proliferation after scratch. A and B, The migration of primary skin fibroblasts from WT mice or SIRT3 KO mice after scratch was photographed at 0, 4, 8 and 12 h. The cell migration rate was quantified as the per cent of migrated area to original area. C, Cellular α‐SMA was stained using Alexa Fluor 488 conjugated IgG (Green) after scratch for 12 h. Bar = 75 µm. D and E, Expression of α‐SMA in mice skin fibroblasts on both mRNA and protein level was detected with real‐time PCR and Western blot respectively after scratch for 12 h. ^*^ *P* < .05, ^**^ *P* < .01 *vs* NG‐stimulated skin fibroblasts from mice with the same genotype, ^#^ *P* < .05, ^##^ *P* < .01 *vs* HG‐stimulated skin fibroblasts from WT mice, n = 6. F, After infection of SIRT3 recombinant adenovirus, the primary skin fibroblasts from SIRT3 KO mice were cultured with high glucose (HG, 30.0 mmol/L). The migration of cells after scratch was photographed at 0, 4, 8 and 12 h. G, SIRT3 expression was measured with Western blot. H, The cell migration rate was quantified as the per cent of migrated area to original area. ^**^ *P* < .01 *vs* HG‐stimulated skin fibroblasts from SIRT3 KO mice with vector infection

Next, we assessed the influence of SIRT3 deficiency on mice skin fibroblast in vitro. The scratch migration assay suggested migration distances after scratch for 4, 8 and 12 hours of mice skin fibroblasts with high‐glucose stimulation was shorter than that with normal glucose exposure in both WT mice and SIRT3 KO mice. Compared to skin fibroblasts from WT mice with high‐glucose stimulation, the migration distance was further shorter than high–glucose‐stimulated skin fibroblasts from SIRT3 KO mice (Figure 5A‐B). The expression of α‐SMA, as a sensitive index for fibroblasts differentiation,^23^ significantly reduced in skin fibroblasts from both WT and SIRT3 KO mice with HG stimulation after scratch for 12 hours. Compared to skin fibroblasts from WT mice with high‐glucose stimulation, α‐SMA expression was further decreased in high–glucose‐stimulated skin fibroblasts from SIRT3 KO mice (Figure 5C‐E). Moreover, migration distance was significantly increased if the recombinant adenovirus carrying the SIRT3 gene was infected into the skin fibroblasts from SIRT3 KO mice (Figure 5F‐H). Taken together, SIRT3 deficiency inhibited high–glucose‐stimulated mice skin fibroblasts migration and proliferation after scratch.

### 3.7 SIRT3 deficiency enhanced ROS but decreased mitochondrial membrane potential in mice skin fibroblasts with high‐glucose stimulation after scratch

**FIGURE 6:**
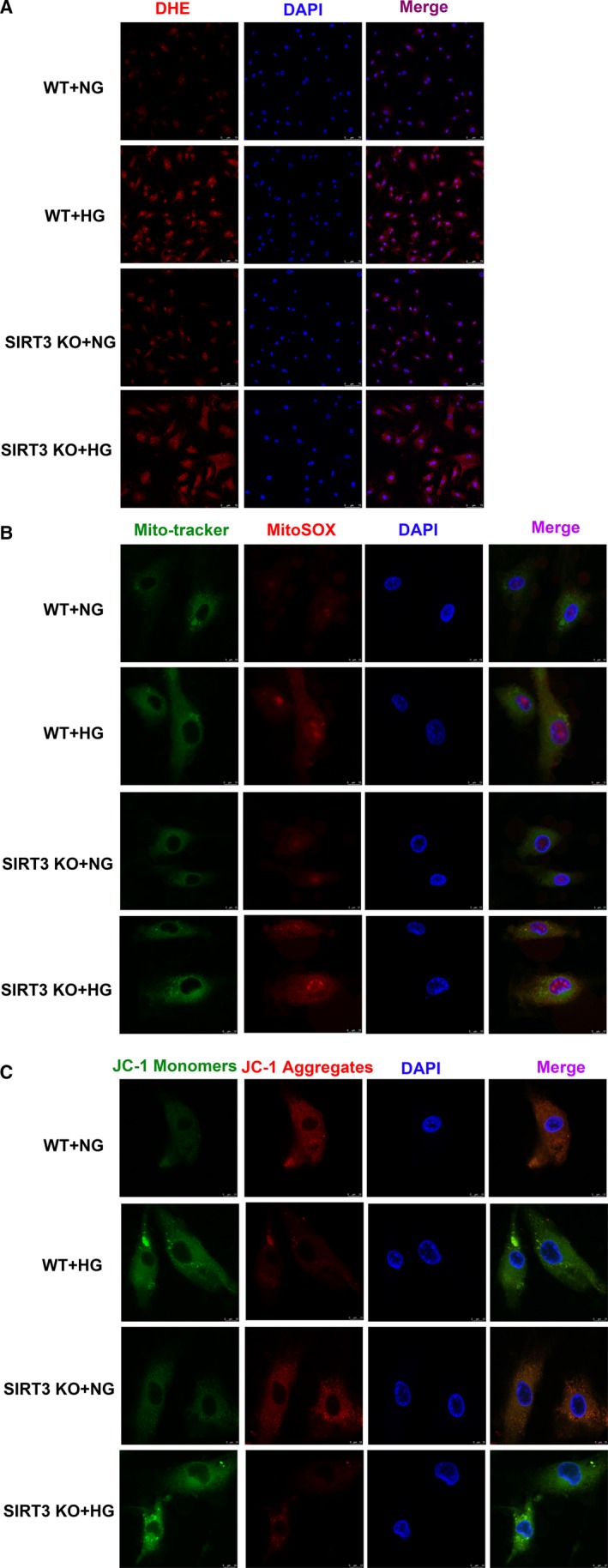
SIRT3 deficiency enhanced ROS in mice skin fibroblasts with high‐glucose stimulation after scratch. A, Global superoxide production in the mice skin fibroblasts after scratch for 12 h was assessed with dihydroethidium (DHE). Bar = 75 μm. B, Mitochondrial reactive oxygen species (ROS) was detected with MitoSOX (Red). Mitochondrial was confirmed by Mito‐tracker (Green). Bar = 10 μm. C, Mitochondrial membrane potential (Δψm) was measured by JC‐1 staining. Bar = 10 μm

Excessive ROS production was considered as one critical mechanism for the delayed wound healing in DM.^7^ Our present study indicated that there was stronger fluorescence intensity of DHE and MitoSOX with HG stimulation, which was further enhanced if SIRT3 was deficient (Figure 6A‐B).

Decrease of mitochondrial membrane potential (Δψm) might contribute to ROS accumulation and cell damage. Our results demonstrated that green fluorescence intensity of JC‐1 monomers, respecting impaired mitochondrial membrane potential, increased after HG stimulation. Meanwhile, red fluorescence intensity of JC‐1 aggregates, respecting normal membrane potential, decreased. Moreover, SIRT3 deficiency further enhanced green but attenuated red fluorescence intensity (Figure 6C). The data suggested that SIRT3 deficiency enhanced ROS but decreased mitochondrial membrane potential in mice skin fibroblasts with high‐glucose stimulation after scratch.

### 3.8 SIRT3 deficiency promoted necroptosis in mice skin fibroblasts with high‐glucose stimulation after scratch

**FIGURE 7:**
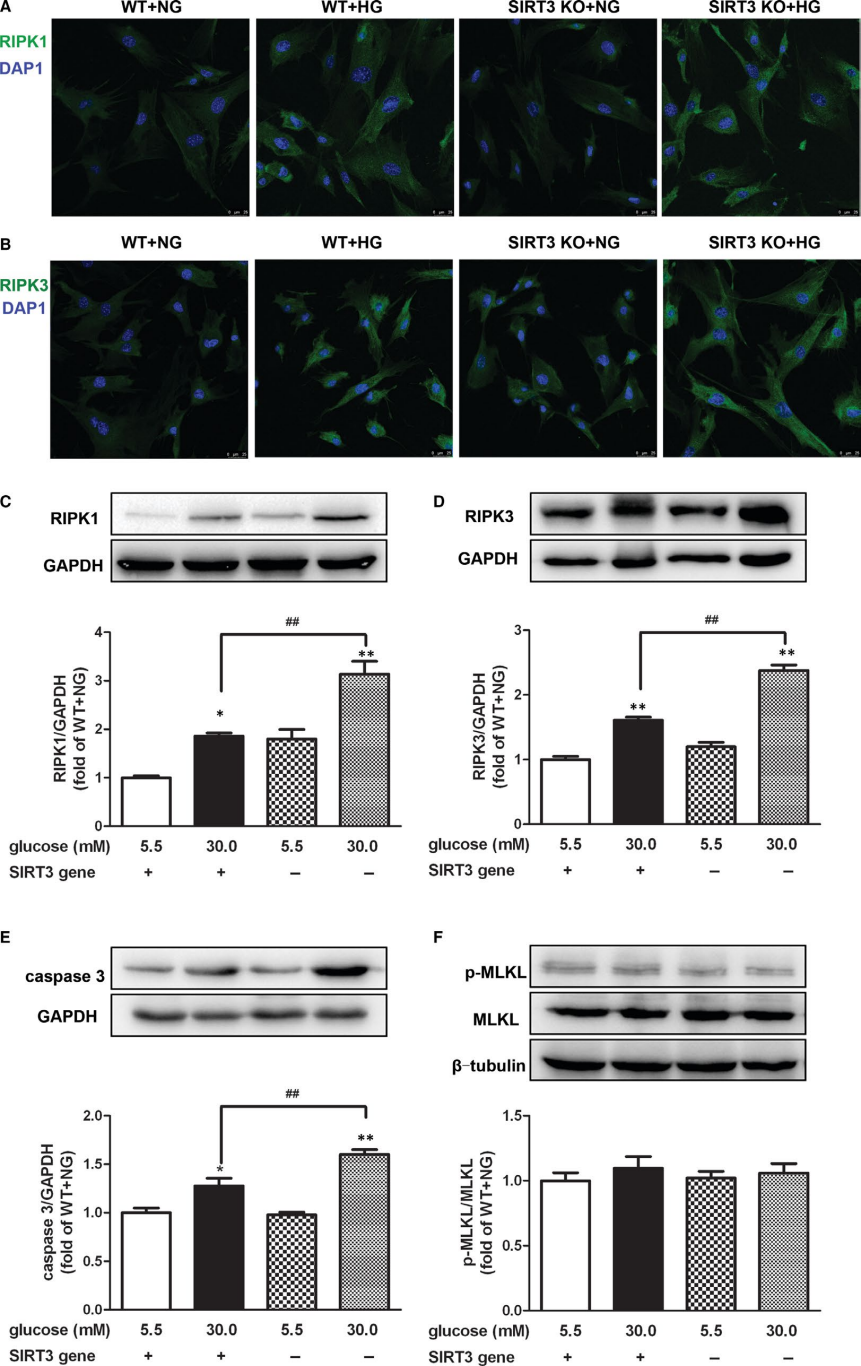
SIRT3 deficiency promoted necroptosis in mice skin fibroblasts with high‐glucose stimulation after scratch. A and B, Cellular RIPK1 and RIPK3 were stained using Alexa Fluor 488 conjugated IgG (Green) after scratch for 12 h. Bar = 25 µm. C‐F, RIPK1, RIPK3, caspase 3, MLKL expression and phosphorylation in mice skin fibroblasts after scratch for 12 h were measured with Western blot. GAPDH or β‐tubulin was serviced as a housekeeping control. *P < .05, **P < .01 *vs* NG‐stimulated skin fibroblasts from mice with the same genotype, ^##^ *P* < .01 *vs* HG‐stimulated skin fibroblasts from WT mice, n = 6

Compared to the normal glucose group, the expression of RIPK1, RIPK3 and caspase 3 was notably increased in the skin fibroblasts from both WT and SIRT3 KO mice with high‐glucose stimulation. Moreover, compared to the high‐glucose stimulation in the skin fibroblasts from WT mice, RIPK1, RIPK3 and caspase 3 expression in skin fibroblasts from SIRT3 KO mice was remarkably increased (Figure 7A‐E), suggesting that SIRT3 played a protective role against necroptosis in mice skin fibroblasts with high‐glucose stimulation after scratch. Interestingly, the p‐MLKL expression showed no statistical significance between normal and high‐glucose stimulation in skin fibroblasts from both WT and SIRT3 KO mice (Figure 7F).

## 4 DISCUSSION

A large number of previous studies have shown that there was low SIRT3 expression in diabetes.^8^, ^26^, ^27^ Several researches have verified that SIRT3 expression was also abnormal in various skin diseases. SIRT3 expression significantly decreased in HaCaT cells with tumour necrosis factor‐α (TGF‐α) stimulation and in the skin of mice or patients with psoriasis.^28^ One group demonstrated that caffeine increased SIRT3 expression to activate autophagy and protect skin from senescence and damage by a free radical inducer in vitro or UV irradiation in vivo.^29^ Biopsy of patients suffering from systemic sclerosis (SSC) indicated that SIRT3 expression in skin significantly decreased compared with the healthy control.^12^ One latest study found that SIRT3 was decreased in wound macrophage via fatty acid‐binding protein 4 up‐regulation.^30^ Our present study also confirmed that that SIRT3 expression in diabetic skin significantly reduced. However, whether decreased SIRT3 expression was critical to contribute to wound healing delaying was unknown. Next, we verified that the skin wound was further delayed in diabetes if SIRT3 was deficient. The migration and proliferation were slower in high–glucose‐stimulated skin fibroblasts from SIRT3 KO mice than that from WT mice. What is more, the speed of mice skin fibroblasts migration was restored after SIRT3 overexpression. Altogether, inadequate SIRT3 is one of the main causes for the delayed healing of diabetic skin wounds.

SIRT3, as a deacetylase in mitochondria, plays an important role in energy metabolism and oxidative stress.^10^, ^31^, ^32^ One study verified that there were more acetylated proteins in the mitochondria in SIRT3 KO mice, which are able to regulate fatty acid import and oxidation, electron transport chain and tricarboxylic acid cycle.^33^ Obviously, most of them are close related to oxidative stress. It was suggested that deacetylation of many oxidative stress‐related proteins in the skin may disorder to produce more oxygen free radicals in the SIRT3 KO mice. Fibroblasts are the main sites for collagen and elastic fibres synthesis. Although skin fibroblasts are located in the dermis under the epidermis, they are more subjected to oxidative damage.^34^ In our experiment, high‐glucose stimulation attenuated proliferation and migration while enhanced oxidative stress in mice skin fibroblasts, which was most serious in that from SIRT3 knockout mice. On the other hand, mitochondria provided a place for ROS generation, and the structural destruction or functional dysfunction was prone to destroy the homeostasis to induce oxidative damage.^35^, ^36^, ^37^ Indeed, we confirmed that SIRT3 KO mice did have above abnormal manifestations on ultrastructure and mitochondrial membrane potential.

The process of skin trauma is essentially the damage by imbalance of skin internal environment with harmful stimulation as oxidative stress, inflammation, radiation etc^23^, ^38^, ^39^, ^40^ During wound healing, various inflammatory cells, such as macrophages, neutrophils, even endothelial cells and fibroblasts, produce active oxygen and free radicals.^41^ Although appropriate amount of free radicals are beneficial to promote wound healing, too much active oxygen suppresses the migration and proliferation of repair cells, inhibits extracellular matrix synthesis and finally delays wound healing.^42^, ^43^ Our study found that the total antioxidant capacity and SOD activity decreased while the MDA level increased in the wound with diabetes, which indicated that the disorder of hyperglycaemia metabolism impaired antioxidant system by weakening the ability of scavenging free radicals and aggravating lipid peroxidation. SIRT3 controls repair‐associated inflammation, which is beneficial to protect against excessive accumulation of ROS.^30^, ^44^ Actually, oxidative stress having been indicated by DHE or MitoSOX staining in diabetes was further strengthened, which might be due to lack of SIRT3 in our study. The enhanced oxidative stress may be the cause of spontaneous ulcer and wound healing delaying in diabetic skin. It was also well acknowledged that defective angiogenesis was also involved in the impaired wound healing in diabetes.^45^, ^46^ In our present study, hyperglycaemia accompanied by SIRT3 lack inevitably led to serve oxidative stress to restrict angiogenic responses including VEGF expression. Accordingly, blood flow around local wound decreased, oxidative stress increased and wound healing delayed after SIRT3 deficiency. These results suggested SIRT3 was involved in limiting ROS generation, boosting angiogenic responses and accelerating wound repair in diabetic individuals.

Several types of harmful stimuli alter the permeability of mitochondrial membrane to reduce membrane potential, block transmission of mitochondrial electron transport chain, suppress cytochrome c production, inhibit adenosine triphosphate (ATP) level, accelerate ROS accumulation, promote local inflammatory cell infiltration and eventually lead to necroptosis.^47^, ^48^ Some studies have confirmed that there was more serious necroptosis in the brain of type 2 diabetic mice than that of non‐diabetic mice.^49^ Necroptosis was also enhanced in cardiomyocytes after high‐glucose stimulation.^50^ Our study found that RIPK1, RIPK3 and caspase 3, but not MLKL, expressions in the skin of diabetic mice significantly increased especially if SIRT3 was absent, suggesting most serious necroptosis in SIRT3 KO mice with diabetes. Above date demonstrated MLKL might not be involved in high–glucose‐induced necroptosis of skin, which was similar to previous finding in HG‐stimulated cardiomyocytes.^50^ Altogether, SIRT3 deficiency was prone to impair mitochondrial structure and function, enhance ROS production, induce necroptosis, attenuate proliferation and repair, and eventually delay skin wound healing.

In conclusion, SIRT3 deficiency delays skin wound healing in diabetes, the mechanism may be related to impaired mitochondria function, enhanced oxidative stress and increased necroptosis. It implicated that SIRT3 may serve as a potentially promising therapeutic target for skin wound healing in diabetes.

## CONFLICT OF INTEREST

The authors confirm that there are no conflicts of interest.

## AUTHORS' CONTRIBUTIONS

Shengju Yang and Mengting Xu carried out most of the experiments; Shengju Yang, Mengting Xu and Guoliang Meng analysed the data; Yan Lu designed the study; Shengju Yang and Yan Lu wrote the manuscript; Shengju Yang and Guoliang Meng provided the statistical support; Yan Lu contributed to the critical revision of article. All authors have read the manuscript and approved the final version.

## Data Availability

The data that support the findings of this study are available from the corresponding author upon reasonable request.
